# Can China’s ecological civilization strike a balance between economic benefits and green efficiency? A preliminary province-based quasi-natural experiment

**DOI:** 10.3389/fpsyg.2022.1027725

**Published:** 2022-10-03

**Authors:** Yushan Li, Baoliu Liu, Pu Zhao, Lin Peng, Zhilin Luo

**Affiliations:** ^1^Research Institute for Eco-Civilization, Chinese Academy of Social Sciences, Beijing, China; ^2^School of Economics and Management, Beijing University of Technology, Beijing, China; ^3^Faculty of Business, Economics and Accountancy, Universiti Malaysia Sabah, Kota Kinabalu, Sabah, Malaysia; ^4^Discipline of International Business, The University of Sydney, Sydney, NSW, Australia; ^5^General Education Faculty, Chongqing Industry Polytechnic College, Chongqing, China

**Keywords:** ecological civilization, total factor productivity, green total factor productivity, economic dividend, green efficiency

## Abstract

To encourage the building of a development route for ecological civilization construction which commensurates with China’s unique national conditions, early demonstration and pilot ecological civilization zones should be built. This study aims to investigate the effects of ecological civilization construction policies on regional total factor productivity, green total factor productivity, and the methods of action by using panel data from 30 provinces in Mainland China from 2005 to 2020. Our findings indicate that the pilot eco-civilization policies have a more significant effect on the promotion of green total factor production, while the effect on total factor productivity is average. Furthermore, the main purpose of the ecological civilization construction pilot is to improve the level of green innovation, optimise the industrial structure and promote the allocation of factors to achieve a win-win situation for regional economic development and green benefits. Moreover, under different levels of economic growth, the pilot eco-civilization policies have a more significant effect on the promotion of green total factor at various stages of economic growth and industrialization. There are also clear discrepancies in how well ecological civilization construction programmes are implemented. Thus, in order to support high-quality regional economic development, it is crucial to continue to advance and promote the pilot eco-civilization initiatives.

## Introduction

Countries and international organisations all over the world have reached an international agreement to support the achievement of the carbon neutrality target and have begun the global process of international climate governance in response to the profound effects of climate change caused by greenhouse gases on human society (Peng et al., 2021; Tan and Wang, 2021; Ding et al., 2022; Wu et al., 2022; Zhao et al., 2022). To reach the Paris Agreement’s 1.5°C temperature rise target, the world must become carbon neutral by the middle of this century (Song et al., 2021; Zhao et al., 2021; Rodrigues et al., 2022). The Intergovernmental Panel on Climate Change (IPCC) reiterated the importance of reducing carbon emissions in its most recent Third Working Group Report of the Sixth Assessment Cycle, recommending that all nations should immediately begin implementing significant reductions to ensure that the temperature rise target will be met.

The goal of China’s creation of an ecological civilization is to decrease carbon emissions, in addition to being a significant approach for combatting climate change (Ahmad and Wu, 2022; Hong et al., 2022; Wang and He, 2022). As a responsible nation, China proposed the idea of energy conservation and emission reduction during the 11th Five-Year Plan and established a binding target for cutting CO_2_ emissions per unit of GDP in each of its three subsequent five-year plans (Su et al., 2020; Zhang S. et al., 2022). At the United Nations General Assembly’s 75th session in September 2020, the Chinese government put forth the targets of carbon peaking by 2030 and carbon neutrality by 2060, fully declaring China’s will as a responsible nation to actively address climate change (Wendebourg, 2020). This reflects China’s role as a responsible country and its proactive approach to addressing climate change in an accurate manner (Filho et al., 2019; Puertas and Marti, 2021; Wang C. et al., 2021). Reaching peak carbon and achieving carbon neutrality have a significant impact on the long-term sustainable development in the region (Chen et al., 2022a; Xu et al., 2022). However, under the impact of the current pandemic, there are concerns on limiting the growth of energy-intensive enterprises in order to reach peak and carbon neutrality, and some regions are making an effort to encourage a rapid resumption to economic growth by continuing to invest in these sectors (Sun et al., 2022). Furthermore, total factor productivity, a key area of supply-side structural reform, has the potential to not only reflect the state of the national economy accurately, but also to significantly contribute to the shift in economic development mode and the closing of the regional economic development gap (Pan et al., 2022).

As a key strategic deployment for the formation of ecological civilization, the pilot ecological civilization zones have taken on the significant responsibility of early and pilot ecological civilization building (Yang and Meng, 2019; Yang Y. et al., 2021). Consequently, the provinces of Fujian, Jiangxi, Guizhou, and Hainan are the focus of this paper’s research. For China to promote high-quality development, it is critical to incorporate carbon emissions into the total factor analysis framework, build green total factor productivity, and analyse the impact of carbon peaking and carbon neutrality on economic development from various perspectives. This is because both ecological civilization building and high-quality economic development are required. Which can be used as a model for developing low-carbon policies that are more sensible and effective in supporting high-quality economic development.

## Literature review

Since the Chinese government put forward the concept of ecological civilization, the main theory of ecological civilization has brought together a wealth of theoretical constructs (Fan et al., 2019; Yin et al., 2019; Ding et al., 2022; Sun et al., 2022; Wu et al., 2022). When it comes to concept development, current mainstream thought tends to identify ecological civilization as a new stage in the evolution of human social civilization from the standpoint of the vertical rise of human historical civilization (Dong et al., 2021; Zuo et al., 2021; Zhang L. et al., 2022). The term ‘ecological civilization’ refers to a relatively recent development in human civilization that has two distinct forms: a basic form based on the achievements of industrial civilization that treats and protects the nature well, and an advanced form that responds to nature, improves human interaction with it, and maintains the ecological operating system (Dong et al., 2021; Tian et al., 2021). The core meaning of the word “ecological civilization” is also investigated, and explained as the relationships between man and nature, the contrast between ecological and modern civilization, and the characteristics of ecological civilization construction throughout the history (Huang and Westman, 2021). An integrated worldview known as ecological civilization takes into account the intricate changes that have occurred in the environment, economics, society, and resource availability (Fernandes and Machado, 2022). The regional system theory of human-earth relationships and the ‘social-economic-natural’ complex ecosystem theory are the foundations of the ecological civilization evaluation system, which offers a thorough and systematic measurement of the level of development of ecological civilization from various perspectives (Zhang et al., 2016, 2017, 2021; Wang D. et al., 2021).

Our findings demonstrate that ecological civilization has varying inclinations to grow both inside and across Chinese regions (Liu et al., 2020; Wang D. et al., 2021). The distribution of natural resources, industrial structure, ecological and cultural awareness, and economic advancement all have an impact on ecological civilization (Franco, 2018; Wang et al., 2020; Li et al., 2022). Other scholars have developed an evaluation index system based on the three elements of pressure-state-response, which describe how people interact with environment around them (Blaison et al., 2019; Wang L. et al., 2021). Some scholars consider the development of ecological civilization as a composite system that can be assessed by choosing assessment indicators for various subsystem levels (Hu et al., 2021; Tan and Wang, 2021; Yang Q. et al., 2021; Zhou and Xu, 2022). By measuring the harm caused by human behavior to the ecological environment and the effectiveness of environmental improvement measures, the latter illustrates the interdependence of ecosystem components, whereas the former analyzes development performance through a multidimensional system of evaluation indicators (Ren et al., 2021; Su et al., 2022).

With the advancement of social development, the concept of giving equal weight to economic development and environmental protection has gained widespread acceptance. By putting the theory of ecological civilization into practise and executing green development with equal emphasis on economic growth and environmental conservation, the tension between economic and social development, resource consumption, and environmental protection may potentially be overcome (Yang Q. et al., 2021; Zhou et al., 2021; Das et al., 2022; Fernandes and Machado, 2022). The nation’s long-term economic prosperity is heavily dependent on how we handle the relationship between man and nature and create a healthy coexistence between man and nature when economic progress is taking place (Xu and Zhong, 2022). Currently, few research has incorporate ecological civilization, green total factor productivity, and total factor productivity into a single analytical framework (Yang Q. et al., 2021; Zhang L. et al., 2022). In order to better understand the scope of the impact of adopting eco-civilization building, this study will develop these three key concepts into an analytical framework.

In comparison to earlier studies, this work adds to existing research in various ways: (1) Beginning with an assessment of the effects of pilot eco-civilization construction policies, this study incorporates eco-civilization construction, total factor productivity, and green total factor productivity into a unified analytical framework, with a focus on whether eco-civilization construction can take into account the win-win enhancement and development of regional economic dividends and green efficiency. (2) The specific effect mechanisms are thoroughly examined, which contributes to the strengthening of the creation of ecological civilization by focusing on specific impact routes. (3) The varied impact of variances in regional economic development levels and stages of industrialization is considered, providing local governments with some reference and experience in creating differentiated policy measures.

## Theoretical analysis and research hypothesis

### Impact of the pilot construction of ecological civilization on regional economic dividends

The development of ecological civilization should support the coordinated expansion of political, economic, cultural, and social construction as a key element of high-quality economic growth (Fan et al., 2019; Zuo et al., 2021; Zhou and Xu, 2022). A major objective in promoting and growing the development of ecological civilization is to achieve win-win development in terms of economic growth and environmental conservation (Xu and Zhong, 2022; Zhou et al., 2022). From the perspective of the goals and the nature of the development of an ecological civilization, it is also an incentive-based method of environmental management, in line with the characteristics of the “compliance cost impact” and the “innovation compensation effect” (Tsang et al., 2021; Ferrara and Giua, 2022; Lian et al., 2022). The pilot development of an ecological civilization demands a considerable capital outlay, which may cause some variations in the regional economy’s efficiency in the short term (Dong et al., 2021; Wang D. et al., 2021; Chen et al., 2022b; Zhang L. et al., 2022). However, the creation of an ecological civilization may unlock economic gains and sustain regional economic growth.

From the aspect of increasing the output of economic advantages, the deployment of eco-civilization pilot projects as an environmental protection method can enhance both regional and enterprise labour productivity (Meng et al., 2021; Tian et al., 2021; Yang Q. et al., 2021). Furthermore, when environmental regulations remain in place for an increasing amount of time, it is easier to determine how this affects the regional economy (Korpela et al., 2020; Patil et al., 2022). Total factor productivity should be increased as a major measure of regional economic efficiency to ensure that all production factors experience acceptable and balanced development. Considering this, the following hypothesis are provided in this study.

*Hypothesis 1a*: The pilot construction of ecological civilization helps to raise the level of regional total factor productivity, which in turn releases the economic dividend effect.

When contemplating the goals of creating an ecological civilization, consideration should be given to enhancing resource efficiency, bolstering ecological environment improvement, and conserving the environment (Peng et al., 2021; Wang and He, 2022). The development of an ecological civilization can reduce environmental input, boost ecological production, and promote regional green growth (Ding et al., 2022). Economic agents prefer lower environmental costs in order to improve regional economic gains. Simultaneously, by creating proper environmental protection measures, it is feasible to mitigate the negative externalities generated by environmental contamination in the manufacturing process (van Balen et al., 2021). The Green Total Factor Productivity (GTP) is a dependable indicator of a region’s capacity for green development and is used to assess a region’s environmental performance (Fang et al., 2022). In general, decreasing carbon emissions boosts a region’s green efficiency. In light of this, this study suggests the following:

*Hypothesis 1b*: The pilot construction of ecological civilization helps to enhance the level of green total factor productivity of a region, which in turn improves the overall green efficiency.

### How ecological civilization pilot projects can benefit local economies and the environment

The successful implementation of pilot eco-civilization projects in China has shown that such programmes not only optimise energy mix and reduce carbon emissions, but also yield environmental advantages (Liu et al., 2018; Hong and Gasparatos, 2020). Increasing regional green innovation levels is the greatest way to ensure low-carbon production by relevant enterprises (Shen et al., 2022). To improve economic development dynamics, regions may be compelled to invest more money in green RandD at the policy level as part of a pilot eco-civilization project (Meng et al., 2021; Yang Q. et al., 2021). The overall capacity for green innovation will be influenced by the high or low conversion rate of innovation outcomes from the standpoints of innovation input and generation (Ding et al., 2022). Therefore, green utility model patents and green invention patents play a key role in achieving a synergistic increase of regional economic dividends and environmental advantages. In light of this, the following hypothesis is advanced:

*Hypothesis 3a*: The prototype construction of an ecological civilization can raise regional total factor productivity as well as green total factor productivity by increasing the degree of green innovation, resulting in the synergistic development of economic benefits and green efficiency.

The government is attempting to control the environment through the eco-civilization pilot project (Zhang et al., 2017; Chen et al., 2022a). To increase the level of advanced industrial structures, it is possible to encourage the transformation and upgrading of industrial structures through the development of ecological civilizations (Dong et al., 2021; Wang D. et al., 2021; Xu et al., 2022). Additionally, it encourages a more logical organization among industries to boost labor productivity in each sector (Ferrara and Giua, 2022). Furthermore, the optimization and modernisation of the industrial structure will help to reduce the number of firms that consume a lot of energy and emit a lot of pollutants, cutting regional carbon emissions, and encouraging low-carbon and environmentally friendly growth in the area (Xu and Zhong, 2022). Based on this, the following hypothesis is proposed in this research.

*Hypothesis 3b*: The prototype ecological civilization can raise regional total factor productivity and green total factor productivity by optimising the industrial structure, resulting in synergistic growth of economic benefits and green efficiency.

The rising and regular flow of factor inputs can aid in the long-term development of the regional economy, which is cited as a crucial component influencing regional economic growth by the Solow model (Topalova, 2010; Yang Q. et al., 2021; Ding et al., 2022; Zhou et al., 2022). On the one hand, ecological civilization has the potential to boost regional total factor production and energy efficiency, which could have positive economic effects (Bournakis and Mallick, 2018; Liu and Xin, 2019; Pan et al., 2022). On the other sides, the increase and flow of regional factor inputs can be increased by improving the ecological environment, thereby realizing the synergistic effect of economic expansion and carbon reduction (Hu et al., 2021; Wang and He, 2022). Hence, optimising factor allocation and adopting cleaner production and living practises are critical to effectively lowering overall regional carbon emissions. This would facilitate the process of constructing an ecological society. Based on this, this paper introduces the following hypothesis.

*Hypothesis 5*: The ecological civilization pilot project can raise regional total factor productivity and green total factor productivity by promoting the level of factor allocation, so realising the synergy between economic benefits and green efficiency.

## Research design

### Multi-period double difference model

Guizhou, Fujian, and Jiangxi were the first three provinces to participate in the establishment of the nation’s ecological civilization pilot zones. The nation later included Hainan to the experimental ecological civilization zone. The implementation of the national pilot ecological civilization zone construction is viewed as a quasi-natural experiment in this paper, with the four provinces of Guizhou, Fujian, Jiangxi, and Hainan serving as the experimental group and the other provinces serving as the control group, to examine the impact of ecological civilization construction on regional total factor productivity and green total want productivity. Using 2016 and 2018 as the time interval points, the multi-period double difference method is used to test whether there is significant variability in total factor productivity and green total factor productivity in the pilot areas while considering the inconsistent timing of the inclusion in the pilot. The creation of the particular multi-period twofold difference model was based on the concept of the Qi et al. (2021) model.


(1) $$\ln y_{it}=\alpha_{0}+\alpha_{1}policy_{it}+\alpha_{1}control_{it}+\mu_{it}+\lambda_{it}+\varepsilon_{it}$$

where *i* and *t* stand for the relevant province, city, and year, respectively, and *y_it_* stands for the size of the respective province, city, and green total factor productivity, *policy_it_* serves as a dummy variable for the pilot eco-civilization building and is a double difference term. *control_it_* designates a number of factors acting as controls on the variables being discussed. μ and λ denote individual fixed effects and year fixed effects, respectively, and ε denotes the random error term.

### Model of the impact mechanism

In this study, the regression mechanism model is further improved in order to more intuitively validate the economic dividend effect and the green efficiency effect produced during the creation of an ecological civilization.


(2) $$\ln Inv_{it}=\theta_{0}+\theta_{1}policy_{it}+\theta_{2}control_{it}+\mu_{i}+\gamma_{t}+\varepsilon_{it}$$

(3) $$\ln Str_{it}=\rho_{0}+\rho_{1}policy_{it}+\rho_{2}control_{it}+\mu_{i}+\gamma_{t}+\varepsilon_{it}$$

(4) $$\ln KA_{it}=\beta_{0}+\beta_{1}policy_{it}+\beta_{2}control_{it}+\mu_{i}+\gamma_{t}+\varepsilon_{it}$$

(5) $$\begin{array}{l} \ln y_{it}=\tau_{0}+\tau_{1}policy_{it}+\tau_{2}Inv_{it}+\tau_{3}Str_{it}+\tau_{4}KA_{it}+\\ \tau_{4}control_{it}+\mu_{i}+\gamma_{t}+\varepsilon_{it} \end{array}$$

where *Inv* represents the level of green innovation and is measured using the total number of green patents by region, *Str* represents the level of industrial structure and is divided into advanced industrial structure (*Ins*) and rationalised industrial structure (*Inu*), and *KA* represents the size of factor allocation and focuses on the influence of labour and capital factors, which is measured using the labour capital stock by region in this paper.

### Variable selection and explanation

Explanatory variables: (1) Total Factor Productivity (TFP) Total factor productivity (TFP) is an important indicator of the effectiveness of economic growth of enterprises. In general, the larger the TFP of a region, the higher the level of regional economic development. To this end, this paper draws on the research methodology of Aigner et al. (1977) and uses stochastic frontier analysis (SFA) and a production function in beyond logarithmic form to measure provincial total factor productivity levels, as follows.


(6) $$\begin{array}{l} \ln P_{it}=\tau_{0}+\tau_{1}InL_{it}+\tau_{2}InK_{it}+\tau_{3}t+1/2\tau_{4}{\left(InL_{it}\right)}^{2}\\ +1/2\tau_{5}{\left(InK_{it}\right)}^{2}+1/2\tau_{6}{\left(t\right)}^{2}+\\ \tau_{7}InK_{it}L_{it}+\tau_{8}tInL_{it}+\tau_{9}tInK_{it}+\varepsilon_{it}-\varphi_{it} \end{array}$$

(7) $$\begin{array}{l} \;\frac{\dot{P}}{P}=\frac{\varphi lnP}{\varphi t}=\frac{\varphi lnf\left(X,t\right)}{\varphi t}+\underset{j}{\sum }\frac{\varphi lnf\left(X,t\right)}{\varphi lnX_{j}}\frac{\varphi lnX_{j}}{\varphi X_{j}}\frac{dX_{j}}{dt}-\\ \frac{\varphi U}{\varphi t}=\frac{\varphi lnf\left(X,t\right)}{\varphi t}+\underset{j}{\sum }\varepsilon_{j}\frac{\dot{X_{j}}}{X_{j}}-\frac{\varphi U}{\varphi t} \end{array}$$

(8) $$\dot{TF}P=SE+TP+\dot{T}E$$

where *P* is real GDP after provincial GDP deflators, *K* is the capital stock, L is the labour force, *ε_it_* represents the random error term and *φ_it_* represents the production inefficiency term. The calculation of capital stock is carried out using the perpetual inventory method. The specific formula is $K_{it}=K_{i,t-1}\left(1-\delta_{t}\right)+I_{it}/R_{it}$ , *K* is the capital stock, *L* is the total regional fixed asset investment, *R* is the price index and I is the depreciation rate, which is taken as 9.6%. In Equation (7) the production function is derived for *t*. In the final result obtained, $\dot{TF}P$ represents the provincial total factor productivity growth rate, *TP* represents the rate of change in production efficiency and $\dot{T}E$ represents the rate of technological progress.

(2) Green Total Factor Productivity (GTFP): a major indicator of a region’s green efficiency, takes into account the impact of environmental issues and focuses on the relationship between economic growth and environmental protection. This work applies the total factor non-radial directional distance function and the SBM-DEA model to calculate the green total factor production level of provinces and municipal regions, with references to Gong and Lin (2018) and Ding et al. (2022).

Core explanatory variable: This study develops policy dummy variables based on national approval of the ecological civilization to reflect the policy consequences brought about by the pilot building of the ecological civilization. It assigns a 1 to provinces that are part of the ecological civilization and a 0 to those that are not.

Control variables: In order to avoid the influence of possible omitted variables and to ensure the accuracy of the regression results, this paper refers to the analysis of Zhang et al. (2014), Bournakis and Mallick (2018), and Liu and Xin (2019), on the factors influencing total factor productivity and green total factor productivity, and selects some of the influencing factors as control variables for the study. (1) urbanisation level (*urban*), the level of urbanisation will affect the economic development of a region, and will also have an important impact on energy saving and emission reduction, this paper uses the proportion of urban population and the total population of each region in that year to measure. (2) The level of upgrading of industrial structure (*ind*), the development of different types of industrial structure will affect the quality development of the region, this paper uses the share of tertiary industry and the total GDP of each region in the current year to measure. (3) The degree of openness to the outside world (*fdi*), the degree of openness to the outside world also has an important impact on regional development, and is measured by the share of foreign direct investment in the year-end GDP of each region. (4) The level of technological innovation (*tec*), which is characterised by the number of patents granted in each region. (5) Level of investment in pollution control (*pollution*), using the share of pollution control investment in GDP at year-end by region. The descriptive statistics of the variables are shown in Table 1.

**Table 1:** Descriptive statistics for variables.

Variables	Sample size	Maximum	Minimum	Mean	SD
*Tfp*	480	2.9001	0.1608	1.5645	0.7534
*Gtfp*	480	4.8889	0.7796	1.5482	0.6768
*Urban*	480	0.8927	0.2950	0.5458	0.1373
*Fdi*	480	0.0868	0.0019	0.0263	0.0019
*Ind*	480	4.1652	0.5447	1.1013	0.5998
*Tec*	480	31.4701	0.0222	3.7483	6.0819
*Pollution*	480	0.0088	0.0002	0.0017	0.0014

### Data sources and descriptive statistics

This paper uses 30 provinces and cities in mainland China (excluding Tibet and Hong Kong, Macao, and Taiwan) from 2005 to 2020 as the sample size, and a small amount of missing data is supplemented by the average growth rate and linear interpolation method. This is due to the difficulty in obtaining some of the data. The majority of the information comes from government websites, including the China Statistical Yearbook, China Energy Statistical Yearbook, China Environmental Statistical Yearbook, and a number of province and city yearbooks. All data were logarithmised before to the regressions in order to ensure the precision of the model regressions and to remove the effects of heteroskedasticity and dimensional issues. Constant prices are treated for index price issues using 2005 as the base period.

## Analysis of empirical results

### Baseline regression results

The Hausman test was used to determine whether a fixed effects or random effects model should be used before conducting the formal regression test. The findings showed that the test was passed, hence, a fixed effects model was selected for the regression. The model should simultaneously take into account both individual effects and time effects due to the panel data sample. The impact of the pilot eco-civilization construction on regional total factor productivity and green total factor productivity was examined using a multi-period twofold difference approach. In the base regression, the effects of the regression with and without the inclusion of control factors were taken into consideration individually. The core explanatory variables and control variables were then combined in the regression model, and the regression results are displayed in Table 2.

Table 2 displays the regression findings for the pilot eco-civilization construction on regional total factor productivity and green total factor productivity. As can be shown, the pilot eco-civilization building can significantly increase the level of regional total factor output because its regression coefficient for the pilot eco-dummy civilization’s variable is significantly positive at the 5% level. The dummy variable’s regression coefficient for green total factor productivity is significantly positive at the 5% level, and a comparison of the corresponding coefficients shows that the pilot eco-civilization construction has a greater positive impact on regional green total factor productivity than total factor productivity. This could be an indication that the development of an ecological civilization can assist both the local economy and environmental efficiency. When control variables are included, the significance of total factor productivity in regression does not change; it is significant at the 10% level, but green total factor productivity is significant at the 1% level. This demonstrates that the ecological civilization pilot project is playing an important role in promoting regional green development. China’s pilot eco-civilization programmes may have a detrimental influence on the region’s short-term economic performance, needing a significant investment in green R&D to repair and protect ecosystems. The long-term goal should thus be to maximise the positive impact of ecological civilization construction on regional environmental performance while also taking advantage of the economic benefits it provides.

**Table 2:** Results of the impact of the eco-civilization pilot on total factor productivity and green total factor productivity.

	(1)	(2)	(3)	(4)
Variables	*Tfp*	*Tfp*	*Gtfp*	*Gtfp*
*Policy*	0.1804^**^ (1.98)	0.3291^*^ (1.80)	0.0389^**^ (2.24)	0.0772^***^ (2.59)
*Urban*		−0.0114^**^ (−2.02)		−0.4631^*^ (−1.65)
*Ind*		0.0744^*^ (1.89)		0.3951^***^ (8.13)
*Fdi*		1.1366^*^ (1.66)		4.3655^**^ (2.40)
*Tec*		0.0035^**^ (2.50)		0.0047^*^ (1.87)
*Pollution*		−0.4980^**^ (−1.98)		−0.2351^**^ (−2.11)
*Constant*	1.5510^***^ (44.39)	1.5906^***^ (8.78)	1.5467^***^ (49.07)	1.2342^***^ (8.07)
Individual fixed	YES	YES	YES	YES
Time fixed	YES	YES	YES	YES
Sample size	480	480	480	480
*R* ^2^	0.8653	0.8678	0.7026	0.6023
*F*	1.6944	0.7478	0.9643	0.9405

***, **, and * denote significant at 1, 5, and 10% significance levels respectively, *t*-values inside brackets.

The level of urbanisation and the amount of investment in pollution control are among the control variables that have a negative impact on the explanatory variables, showing that it is important to prevent resource waste and the irrational allocation of resource caused by excessive urban expansion when promoting urbanisation. In order to maximise the use of investment funds, consideration should also be given to increasing the effectiveness of investments in pollution control. Regional and green total factor production will benefit from the degree of industrial structure modernization, openness to the outside world, and technological innovation. This further demonstrates the significance of boosting technological innovation and industrial structure optimization to support high-quality regional economic development.

### Parallel trend test

When evaluating the pilot eco-civilization construction’s effects on regional total factor productivity and green total factor productivity using the double difference method, it is important to take into account whether the explanatory variables in the chosen experimental and control groups satisfy the parallel trend setting. This study takes into consideration the choice of 2016 and 2018 as the policy implementation years as well as the fact that 2016 is the year in which the policy commenced as it chooses to include whether or not to carry out the development of a pilot eco-civilization region as a policy dummy variable. In order to assess the size of the significance level of the variables in various years, this work used 2016 as the year for the regression test and used the first three and final 4 years for the assignment research. The regression results of the parallel trend test are displayed in Tables 3, 4. It is clear that the regression coefficients for the dummy variables were not significant before to the ecological civilization pilot policy’s introduction but increased in significance in the years that followed, supporting the validity of the twofold difference model.

**Table 3:** Regression results of the total factor productivity parallel trend test.

Pilot time	(1)	(2)	(3)	(4)	(5)	(6)	(7)
	Before 1	Before 2	Before 3	After 1	After 2	After 3	After 4
Policy	0.1089 (1.04)	0.1788 (1.08)	0.0511 (0.48)	0.4015^*^ (1.72)	0.5066^*^ (1.67)	0.6489^***^ (2.70)	0.6181^***^ (2.69)
Control Variables	YES	YES	YES	YES	YES	YES	YES
Time fixed	YES	YES	YES	YES	YES	YES	YES
Individual fixed	YES	YES	YES	YES	YES	YES	YES
*N*	480	480	480	480	480	480	480
*R* ^2^	0.8659	0.8042	0.7943	0.8563	0.8574	0.8423	0.8042

***, **, and * denote significant at 1, 5, and 10% significance levels respectively, *t*-values inside brackets.

**Table 4:** Regression results of the green total factor productivity parallel trend test.

Pilot time	(1)	(2)	(3)	(4)	(5)	(6)	(7)
	Before 1	Before 2	Before 3	After 1	After 2	After 3	After 4
Policy	0.1023 (1.18)	0.1375 (0.81)	0.0933 (1.15)	1.3545^*^ (1.95)	0.5048^***^ (2.75)	0.4132^**^ (2.43)	0.1375^*^ (1.81)
Control variables	YES	YES	YES	YES	YES	YES	YES
Time fixed	YES	YES	YES	YES	YES	YES	YES
Individual fixed	YES	YES	YES	YES	YES	YES	YES
*N*	480	480	480	480	480	480	480
*R* ^2^	0.1109	0.1259	0.1240	0.1935	0.3601	0.3264	0.3102

***, **, and * denote significant at 1, 5, and 10% significance levels respectively, *t*-values inside brackets.

### Robustness tests

The main regression in this study is conducted using the twofold difference approach, which might somewhat reduce the impact of time variation. However, sample selection bias and its effects on the reliability of the regression results must still be taken into account. Therefore, this study selects a suitable method to carry out robustness tests in order to prevent the issue of error in the model regression.

#### Placebo test

The placebo test is used to evaluate how much the event affected the sample. Therefore, this paper makes reference to Topalova (2010) research strategy of conducting a placebo test on the pre-event sample in order to guarantee the validity of the study results. If the regression results were not significant, it indicated that the factors affecting regional total factor productivity and green total factor productivity were in fact caused by the event of carrying out the ecological civilization construction. In this paper, the multi-period double difference method was used, and 2016 and 2018 were taken as the time years of the implementation of the ecological civilization construction. In order to achieve this, the year 2012, which marked the start of the eco-civilization construction, was selected as the dividing point, making the years 2010 and 2011 the pre-implementation years and the years 2012 and 2013 the post-implementation years. Regression results are shown in Table 5 below.

**Table 5:** Placebo test regression results.

	(1)	(2)
**Variables**	***tfp***	***gtfp***
*Policy*	0.1561 (1.25)	0.0704 (0.94)
*Urban*	−1.0721^**^ (−2.24)	−0.5360^**^ (−2.36)
*Ins*	0.1728^**^ (2.03)	1.8736^***^ (7.57)
*Fdi*	0.5750^*^ (1.67)	1.1920^**^ (2.10)
*Tec*	0.0152^*^ (1.72)	0.1294^***^ (3.54)
*Pollution*	−0.6595^*^ (−1.84)	−0.4793^*^ (−1.71)
Constant	2.6158^***^ (3.22)	−7.9641^**^ (−2.32)
Individual fixed	YES	YES
Time fixed	YES	YES
Sample size	120	120
*R* ^2^	0.7508	0.5954

***, **, and * denote significant at 1, 5, and 10% significance levels respectively, *t*-values inside brackets.

When the placebo test is conducted for the time period prior to the event of carrying out the ecological civilization construction, it can be seen from the regression results of the test that the regression coefficients of the policy dummy variables are not significant in the regression results for the total factor productivity and green total factor productivity changes, which further indicates that carrying out the ecological civilization construction will have a positive impact. This further implies that the eco-civilization pilot project’s implementation will have an impact on the scope of regional total factor productivity and green total factor productivity, verifying the accuracy of the study’s findings.

### Analysis of the impact mechanism

The influence of pilot eco-civilization development on regional total factor productivity and green total factor productivity has been extensively examined in the prior research, but it is still necessary to further understand the underlying impact mechanisms. According to the theoretical analysis and research hypothesis, the pilot construction of ecological civilization can link ecological environmental protection and economic development effectively, and as a result, fully exploit the spillover effects brought about by the pilot construction process, such as the improvement of regional green innovation capacity and the rationalisation of industrial structure development, thus providing a new development pathway for achieving. Tables 6, 7 display the precise regression results for the impact mechanism effects.

**Table 6:** Results of the impact mechanism test (1).

Variables	Green Innovation			Element configuration		
	*Inv*	*tfp*	*gtfp*	*KA*	*tfp*	*gtfp*
	(1)	(2)	(3)	(4)	(5)	(6)
Policy	0.0815^**^ (2.01)	0.1205^*^ (1.78)	0.2540^***^ (2.89)	1.0152^**^ (2.18)	0.1520^**^ (2.40)	0.1214^**^ (2.09)
Med		0.2443^**^ (2.20)	0.0842^**^ (1.98)		0.1205^**^ (2.32)	0.0843^*^ (1.90)
Urban	−0.0248^*^ (−1.75)	−0.1529^**^ (−2.08)	−0.2039^*^ (−1.85)	−0.1053^*^ (−1.82)	−0.0541^**^ (−2.24)	−0.0412^*^ (−1.70)
Ind	0.5230^**^ (2.12)	1.0478^**^ (2.30)	0.6239^*^ (1.82)	1.0320^*^ (1.75)	0.8120^**^ (2.44)	1.0623^**^ (2.18)
Fdi	0.1412^*^ (1.85)	0.3206 (1.54)	0.2683^**^ (2.05)	0.0842^**^ (2.10)	0.1419^**^ (2.15)	0.3108^**^ (2.05)
Tec	0.4247^**^ (2.05)	0.1248^*^ (1.74)	0.3011^*^ (1.84)	0.1547^**^ (2.15)	0.0408^**^ (2.34)	0.1218^**^ (2.10)
Pollution	−1.0206^**^ (−2.17)	−0.8240^**^ (−1.99)	−0.0563^**^ (−2.01)	−0.2167^*^ (−1.81)	−0.0562^**^ (−2.24)	−0.0310^**^ (−2.30)
Constant	0.4127^**^ (2.43)	0.2517^***^ (2.62)	0.0271^**^ (2.42)	0.6129^***^ (3.18)	0.1606^***^ (4.20)	0.0227^**^ (2.35)
Individual fixed	YES	YES	YES	YES	YES	YES
Time fixed	YES	YES	YES	YES	YES	YES
*N*	480	480	480	480	480	480
*R* ^2^	0.5362	0.5203	0.6301	0.5903	0.6012	0.6525

***, **, and * denote significant at 1, 5, and 10% significance levels respectively, *t*-values inside brackets.

**Table 7:** Results of the impact mechanism test (2).

Variables	*Ins*			*Inu*		
	*Inv*	*Tfp*	*Gtfp*	*KA*	*Tfp*	*Gtfp*
	(7)	(8)	(9)	(10)	(11)	(12)
*Policy*	0.0642^**^ (2.10)	0.1202^**^ (2.24)	0.1867^***^ (2.82)	0.2310^**^ (2.18)	0.0941^*^ (1.90)	0.2041^*^ (1.85)
*Med*		0.0726^*^ (1.89)	0.0152^**^ (2.03)		0.0426^**^ (2.22)	0.0360^**^ (2.27)
*Urban*	−0.0248^*^ (−1.75)	−0.1529^**^ (−2.08)	−0.2039^*^ (−1.85)	−0.1053^*^ (−1.82)	−0.0541^**^ (−2.24)	−0.0412^*^ (−1.70)
*Ind*	0.5230^**^ (2.12)	1.0478^**^ (2.30)	0.6239^*^ (1.82)	1.0320^*^ (1.75)	0.8120^**^ (2.44)	1.0623^**^ (2.18)
*Fdi*	0.1412^*^ (1.85)	0.3206 (1.54)	0.2683^**^ (2.05)	0.0842^**^ (2.10)	0.1419^**^ (2.15)	0.3108^**^ (2.05)
*Tec*	0.4247^**^ (2.05)	0.1248^*^ (1.74)	0.3011^*^ (1.84)	0.1547^**^ (2.15)	0.0408^**^ (2.34)	0.1218^**^ (2.10)
*Pollution*	−1.0206^**^ (−2.17)	−0.8240^**^ (−1.99)	−0.0563^**^ (−2.01)	−0.2167^*^ (−1.81)	−0.0562^**^ (−2.24)	−0.0310^**^ (−2.30)
Constant	0.4127^**^ (2.43)	0.2517^***^ (2.62)	0.0271^**^ (2.42)	0.6129^***^ (3.18)	0.1606^***^ (4.20)	0.0227^**^ (2.35)
Individual fixed	YES	YES	YES	YES	YES	YES
Time fixed	YES	YES	YES	YES	YES	YES
*N*	480	480	480	480	480	480
*R* ^2^	0.5362	0.5203	0.6301	0.5903	0.6012	0.6525

***, **, and * denote significant at 1, 5, and 10% significance levels respectively, t-values inside brackets.

The regression findings of the influence mechanism’s effect are displayed in Tables 6, 7. After taking into account factor allocation, sophisticated and rationalised industrial structure, and green innovation, it can be seen that the related regression coefficients are noticeably positive. This suggests that the ecological civilization’s pilot project will strengthen its impact *via* these routes. In particular, for total factor productivity at the 10% level and for green total factor productivity at the 1% level, the regression coefficients for green innovation are significantly positive. This shows that, at this point, enhancing regional green innovation is more likely to have a substantial impact on regional green development while still needing to be enhanced is the role of improving regional economic efficiency. Second, from the standpoint of factor allocation, both the total factor productivity and green total factor productivity regression coefficients are significantly positive at the 5% level, supporting the critical role that optimising factor resource allocation plays in the development of ecological civilization.

The regression coefficients of the policy dummy variables are more significant under the influence of an advanced industrial structure from the standpoint of an advanced and rationalised industrial structure. This shows that the pilot development of ecological civilization is more conducive to the optimization and upgrading of industrial structure in the short term, but the rationalisation of inter-industrial structure allocation still requires more development. The aforementioned regression results further support the earlier research premise that the pilot development of an ecological civilization can, through a variety of mechanisms, increase regional total factor productivity and green total factor productivity.

### Heterogeneity analysis

#### Considering differences in the level of economic development

This research further separated the sample into eastern, central, and western areas to undertake validation estimation, taking into account that variations in the level of economic development of different regions would have an impact on the regression results. Table 8 displays the specific estimation results.

**Table 8:** Regression results for the effects of different levels of economic development.

Variables	Eastern		Middle		Western	
	*Tfp*	*Gtfp*	*Tfp*	*Gtfp*	*Tfp*	*Gtfp*
Policy	0.6305^**^ (2.13)	1.0361^***^ (3.24)	0.1810^**^ (2.32)	0.2319^**^ (2.19)	0.0846^*^ (1.92)	0.2149^*^ (1.86)
Control variables	YES	YES	YES	YES	YES	YES
Time fixed	YES	YES	YES	YES	YES	YES
Individual fixed	YES	YES	YES	YES	YES	YES
Constant	0.2740^***^ (3.26)	2.2361^***^ (4.10)	1.6250^**^ (2.12)	3.0102^**^ (4.64)	2.1245^***^ (3.12)	1.1245^***^ (2.80)
*R* ^2^	0.7203	0.7310	0.7012	0.6920	0.6512	0.6302
*N*	192	192	144	144	144	144

***, **, and * denote significant at 1, 5, and 10% significance levels respectively, *i*-values inside brackets.

The findings in Table 8 demonstrate that the effects of ecological civilization on various geographical areas varied significantly. The pilot development of an ecological civilization can more quickly show the economic benefits it offers and its impact on the improvement of the ecological environment for the eastern areas, i.e., the rapid increase in total factor productivity and green total factor productivity levels. In contrast, the middle and western regions have better resource endowments because of their geographic location and lower levels of foreign investment, but in the short term, they still need to put forth significant effort to increase total factor productivity and green total factor productivity to ensure that the development of ecological civilization can play a more advantageous role in promoting it.

#### Considering differences in the stage of industrialisation development

In addition to being strongly tied to the stage of economic growth in which an area is located, the development of ecological civilization may also be related to the rate at which the industrialization process is progressing. Therefore, the research sample is separated into three periods in this paper based on Chinnery’s theory of industrial staging, and panel data of regions at various phases of industrialization development are estimated. The regression findings are displayed in Table 9.

**Table 9:** Regression results for the impact of different stages of industrialisation development.

Variables	Post-industrialisation		Late industrialisation (second half)		Late industrialisation (first half)	
	*Tfp*	*Gtfp*	*Tfp*	*Gtfp*	*Tfp*	*Gtfp*
Policy	0.3024^***^ (2.62)	0.8105^***^(2.83)	1.0240^**^ (2.28)	0.0634^***^ (2.75)	0.7810^*^ (1.92)	0.5104^**^ (1.98)
Control variables	YES	YES	YES	YES	YES	YES
Time fixed	YES	YES	YES	YES	YES	YES
Individual fixed	YES	YES	YES	YES	YES	YES
Constant	0.5769^**^ (2.43)	1.0219^***^ (2.92)	1.6342^***^ (3.30)	2.1204^***^ (3.26)	0.6040^***^ (4.10)	0.8163^**^ (2.36)
*R* ^2^	0.6402	0.6230	0.6012	0.5923	0.5521	0.5362
*N*	144	144	112	112	224	224

***, **, and * denote significant at 1, 5, and 10% significance levels respectively, *t*-values inside brackets.

The regression results in Table 9 demonstrate that the regression coefficients of the policy dummy variables are all significantly positive at the 1% level for regions in the post-industrialization stage, demonstrating that by this period, both factor resources and technological innovation strength have significantly improved because the level of industrialization is already at a higher status. In contrast, regions in the late industrialization (second half) and late industrialization (first half) stages still need to raise their level of innovation in science and technology, abandon the use of traditional, high-energy industries as growth engines, and make sure that the ecological civilization’s construction can better support the high-quality development of the regional economy.

## Conclusions and policy recommendations

### Research conclusions

The purpose of this study is to examine the impact of China’s ecological civilization construction strategy on regional total factor production and green total factors. A multi-period twofold difference approach is developed. Several robustness tests have been run to determine whether the implementation of the eco-civilization pilot project can accomplish win-win enhancement and the growth of regional economic dividends and green efficiency. The analysis of its specific mechanisms of action, which takes into consideration the diverse impacts of various regional levels of economic growth and stages of industrialization development, leads to the following key findings.

To begin with, while the contribution to total factor productivity is minor, the execution of eco-civilization construction initiatives has a greater impact on regional green total factor production. Second, according to the analysis of specific impact pathways, the ecological civilization construction implementation can boost regional total factor productivity and green total factor productivity by increasing the level of green innovation, improving the industrial structure, and encouraging the level of factor allocation. The pilot eco-civilization program has a general impact on industrial structure rationalization and a more obvious impact on the promotion of advanced industrial structure. Finally, the heterogeneity analysis results show that in the post-industrialization period, it is easier for the eastern regions and provinces to carry out pilot eco-civilization construction to promote regional economic efficiency and emission reduction capacity, i.e., the promotion effect on regional total factor productivity and industrialization stage development.

### Policy recommendations

Based on the above research findings, this paper puts forward the following relevant policy recommendations:

Firstly, the development of an ecological civilization should be consistently encouraged and enhanced, taking into account the beneficial experience gained during the construction of the four pilot provinces of Guizhou, Jiangxi, Fujian, and Hainan. The creation of pilot ecological civilization zones is being done in order to continuously explore and create a development model of ecological civilization construction fit for China’s national conditions. The non-pilot provinces should fully learn and absorb them in order to develop an ecological civilization development path with local characteristics, while the pilot provinces should fully summarize and share their experiences and lessons learned in the process of carrying out the pilot construction of ecological civilization, distill the successful paths and promote them.

Secondly, it should boost support for green R&D, boost the effectiveness of green patent conversion, and encourage the wise use of factor resources. Ensure the balanced development of inter-industry structures in order to achieve the goal of realizing the financial gains and environmental advantages brought about by the process of building an ecological civilization, as well as fostering the growth of regional total factor productivity and green total factor productivity.

Thirdly, it is important to concentrate on the influence of heterogeneous factors, to design and implement differentiated policy combinations for different regions, and to apply policies according to local conditions in order to ensure that the outcomes of ecological civilization construction can be enriched. This is necessary in order to fully play out the positive impact of the pilot construction of ecological civilization on society, the economy, and the ecological environment.

### Research deficiencies and prospects

Based on provincial-level data, this paper analyzes the impact and mechanism of China’s ecological civilization construction on regional economic dividends and green efficiency. In the follow-up research, further research can be carried out from the data at the prefecture-level city level.

## Data availability statement

The original contributions presented in the study are included in the article/Supplementary material, further inquiries can be directed to the corresponding authors.

## Author contributions

BL, PZ, LP, and ZL performed material preparation, data collection and analysis. YL wrote the first draft of the manuscript. All authors contributed to the article and approved the submitted version.

## Funding

YL acknowledges the financial support from postdoctoral innovation project of Chinese Academy of Social Sciences “Research on Beijing Winter Olympics and Urban Sustainable Development.” BL acknowledges the financial support from National Natural Science Foundation of China Key Project “Research on the Construction of China’s Economic Transformation Model for Carbon Neutrality” (TZ140001).

## Conflict of interest

The authors declare that the research was conducted in the absence of any commercial or financial relationships that could be construed as a potential conflict of interest.

## Publisher’s note

All claims expressed in this article are solely those of the authors and do not necessarily represent those of their affiliated organizations, or those of the publisher, the editors and the reviewers. Any product that may be evaluated in this article, or claim that may be made by its manufacturer, is not guaranteed or endorsed by the publisher.
